# Online Social Environment Impacts Feelings of Social Connection in Older Adults During the COVID-19 Pandemic

**DOI:** 10.1093/geroni/igaa057.3491

**Published:** 2020-12-16

**Authors:** Brittany Derynda, Mary Goodyear, Jade Kushner, Nicole Cook

**Affiliations:** 1 Nova Southeastern University Dr. Kiran C Patel College of Osteopathic Medicine, Clearwater, Florida, United States; 2 Nova Southeastern University Dr. Kiran C Patel College of Osteopathic Medicine, Fort Lauderdale, Florida, United States; 3 College of Psychology, Fort Lauderdale, Florida, United States; 4 Nova Southeastern University, Davie, Florida, United States

## Abstract

Evidence suggests that nearly one-third of older adults experience loneliness and/or social isolation; an increase in these rates during the current pandemic is anticipated. The Lifelong Learning Institute (LLI) at Nova Southeastern University (NSU) in South Florida has worked to engage seniors in fun learning activities and social opportunities since 1977. When “stay at home” orders went into effect in March, 2020 the LLI moved to online program delivery via Zoom. To understand the implementation of zoom among LLI members, NSU students, researchers and LLI member advisory committee developed a cross-sectional research study using an online survey that was administered to LLI members three months post-zoom implementation in May, 2020. Results among the 127 responders demonstrated that a majority of members were not comfortable using zoom (57%) especially the chat, reactions or camera features. More than 80% of responders did report that zoom helped them keep their spirits up. Respondents had specific feedback to improve Zoom programming including Youtube videos on use, retraining, training on features (e.g. chat, camera, reaction), closed captioning, program reminders and links sent out more frequently and within 30 minutes of start time. There were also several comments about internet connectivity, identifying opportunities for router and internet plan education. Finally, respondents noted new opportunities to enhance virtual programming including engaging speakers from across the nation and world. In summary, direct feedback from seniors on how to improve the online social and learning environment is pivotal to improving experience, programming and social connection during COVID-19.

